# Rate of vertical transmission of human papillomavirus from mothers to infants: Relationship between infection rate and mode of delivery

**DOI:** 10.1186/1743-422X-9-80

**Published:** 2012-04-12

**Authors:** Hyun Park, Si Won Lee, In Ho Lee, Hyun Mee Ryu, A Reum Cho, Young Soon Kang, Sung Ran Hong, Sung Soon Kim, Seok Ju Seong, Son Moon Shin, Tae Jin Kim

**Affiliations:** 1 Comprehensive Gynecological Cancer Center, CHA Bundang Medical Center, CHA University, College of Medicine, Bundang, South Korea; 2 Department of Obstetrics & Gynecology, Cheil General Hospital and Women's Healthcare Center, Kwandong University, College of Medicine, Seoul, South Korea; 3 Laboratory of Molecular Oncology, Cheil General Hospital and Women's Healthcare Center, Kwandong University, College of Medicine, Seoul, South Korea; 4 Laboratory of Research & Development for Genomics, Cheil General Hospital and Women's Healthcare Center, Kwandong University, College of Medicine, Seoul, South Korea; 5 Department of Pathology, Cheil General Hospital and Women's Healthcare Center, Kwandong University, College of Medicine, Seoul, South Korea; 6 Division of AIDS, Korea Centers for Disease Control and Prevention, Korea National Institute of Health, Seoul, South Korea; 7 Department of Obstetrics & Gynecology, CHA Gangnam Medical Center, CHA University, College of Medicine, Seoul, South Korea; 8 Department of Pediatrics, Cheil General Hospital and Women's Healthcare Center, Kwandong University, College of Medicine, Seoul, South Korea

## Abstract

**Background:**

In contrast to consistent epidemiologic evidence of the role of sexual transmission of human papillomavirus (HPV) in adults, various routes may be related to HPV infection in infants. We have assessed the extent of HPV infection during the perinatal period, and the relationship between mode of delivery and vertical transmission.

**Results:**

A total of 291 pregnant women over 36 weeks of gestation were enrolled with informed consent. Exfoliative cells were collected from maternal cervix and neonatal buccal mucosa. HPV infection and genotypes were determined with an HPV DNA chip, which can recognise 24 types. The HPV-positive neonates were re-evaluated 6 months after birth to identify the presence of persistent infection. HPV DNA was detected in 18.9 % (55/291) of pregnant women and 3.4 % (10/291) of neonates. Maternal infection was associated with abnormal cytology (p = 0.007) and primiparity (p = 0.015). The infected neonates were all born to HPV-positive mothers. The rate of vertical transmission was estimated at 18.2 % (10/55) which was positively correlated with maternal multiple HPV infection (p = 0.003) and vaginal delivery (p = 0.050), but not with labour duration and premature rupture of membranes. The rate of concordance of genotype was 100 % in mother-neonate pairs with vertical transmission. The neonatal HPV DNAs found at birth were all cleared at 6 months after delivery.

**Conclusions:**

Vertical transmission of HPV DNA from HPV infected mother to the neonate increased when the infant was delivered through an infected cervix. However, the absence of persistent infection in infants at 6 months after delivery may suggest temporary inoculation rather than true vertical infection.

## Background

Approximately 200 different genotypes of human papillomavirus (HPV) have been identified and >40 types are associated with anogenital diseases [1]. While these mucosal types are suspected of affecting predominantly adults, reports of HPV related diseases in the oropharyngeal and anogenital mucosa of infants and children born to HPV infected mothers are increasing [2].

In contrast to the consistent epidemiologic reports, including the role of sexual transmission of HPV in adults [3], potential routes of HPV infection from mother to newborns were as follows: 1) during passage of the fetus through an infected birth canal, 2) ascending infection after premature rupture of the membranes, 3) infected sperm at fertilization, and 4) hematogenous spread [4].

The extent and risk factors of HPV infections in infants have been controversial. For pregnant women and their babies, the only meta-analysis to date show that vertical transmission developed in one-third of neonates born to infected mothers mainly through vaginal deliveries [5]. However, there was a wide variation in the rate of vertical transmission ranging from 0-80 % [4,6-9]. Furthermore, a report with type-specific polymerase chain reaction (PCR) or DNA sequencing has identified the lack of concordance of HPV genotypes in 57-69 % of the mother-neonate pairs [2]. The discrepancy is regarded as evidence proposing various routes of viral transmission other than the birth canal.

HPV DNA chip and PCR-based oligonucleotides microarray have been useful in many screening programs for the early diagnosis of cervical dysplasia and cancer [10]. This technique can accurately determine HPV-positivity and distinguish types of HPV DNA, even in cases infected with multiple types [11]. In addition to potential factors associated with neonatal infection, HPV genotying can be helpful in explaining the mechanism of viral transmission.

In this study, we have determined the prevalence of HPV infection in pregnant women and the rate of vertical transmission during the perinatal period. The risk factors associated with viral transmission were also explored, including HPV genotypes.

## Results

A total of 300 term pregnant women were enrolled over the study period. Analyses were restricted to 291 women who gave birth in the institutes through vaginal delivery (n = 193, 66.3 %) and caesarean section (n = 98, 33.7 %).

### Maternal HPV status

The mean age of pregnant women was 32.8 years and the prevalence of HPV infection was 18.9 % (55/291) in maternal cervical swabs at enrolment determined by means of HPV DNA chips. In the cytology performed at the first trimester, 283 women were negative for intraepithelial lesions and 8 had atypical squamous cells of undetermined significance. Women with abnormal cytology were referred for colposcopic examination and the results indicated no dysplasia in the cervix. Prevalence of HPV infection in the newborns was 3.4 % (10/291) from swabs of oral mucosa at delivery.

Details of the maternal characteristics regarding age, gestational history, bacterial genital infection, gestational diabetes mellitus, and abnormal cytology are given in Table 1. Women with primiparity (p = 0.015) or abnormal cytology found at the first trimester (p = 0.007) showed a higher prevalence of HPV infection in univariate analyses with a chi-square or Fisher’s exact test. These associations were not confounded by age, when adjusted using the Mantel-Haenszel test for stratified analysis. HPV infection was less frequent in multiparous women (p = 0.034) and more in women with abnormal cytology (p = 0.011), regardless of age.

**Table 1:** Characteristics associated with maternal HPV status

**Characteristics**		**HPV(+)**	**(%)**	**HPV(−)**	**(%)**	P-value*
Age (y)	≤30	21	(24.4)	65	(75.6)	0.252
	31-35	20	(15.4)	110	(84.6)	
	≥36	14	(18.7)	61	(81.3)	
Gravida	1	25	(18.8)	108	(81.2)	0.803
	2	20	(20.6)	77	(79.4)	
	≥3	10	(16.4)	51	(83.6)	
Para	0	44	(22.9)	148	(77.1)	0.015
	≥1	11	(11.1)	88	(88.9)	
Abortion	0	31	(16.1)	161	(83.9)	0.095
	≥1	24	(24.2)	75	(75.8)	
Bacterial genital infection	(−)	54	(18.8)	234	(81.3)	0.468
	(+)	1	(33.3)	2	(66.7)	
GDM	(−)	55	(19.4)	229	(80.6)	0.354
	(+)	0	(0.0)	7	(100.0)	
Abnormal cytology	(−)	50	(17.7)	233	(82.3)	0.007
	(+)	5	(62.5)	3	(37.5)	

GDM: gestational diabetes mellitus, *P < 0.05 indicates statistical significance by a chi-square or Fisher’s exact test.

Among the types specified by HPV DNA chip, HPV-16 was the most frequent type in the maternal cervix (n = 55), comprising 8 cases (14.5 %) and other specified HPV types were as follows: HPV-6, -56 each in 5 cases (9.1 %); -53, -58, -70 each in 4 cases (7.3 %); -39, -40 each in 3 cases (5.5 %); and −44, -51, -31 each in 2 cases (3.6 %). Each of HPV-11, -18, -33, -35, -45, and −66 was noted only in one participant. HPV-other type, which was positive in PCR but negative in HPV DNA chip, was noted in 29.1 % (16/55). Genotyping showed that 58.2 % (32/55) were infected with at least one type of high-risk HPVs.

Co-infection with multiple genotypes was noted in 8 mothers; 3 mothers were infected with 3 types, and 5 with 2. HPV-53 (n = 4) was the most frequent type found in multiple infection. HPV-40 was detected in 3 cases and HPV-44 and −56 were detected in 2 cases. HPV-11, -16, -33, -35, -58, -66 and −68 were detected once.

### Neonatal HPV status

At birth, HPV DNA was detected in 10 (3.4 %, 10/291) neonates who were born to HPV-positive mothers. There was no infection among infants born to HPV-negative mothers. Therefore the overall frequency of HPV transmission from HPV-positive mothers to their infants was 18.2 % (10/55). Genotypes were concordant in all of the mother-neonate pairs (Table 2). If one type of HPV was identical among multiple types detected in a mother neonate pair, the case was considered to be concordant. HPV-16 and HPV-53 were the most frequent types and each was noted in 2 newborns. For these 10 HPV-positive newborns, HPV DNA chip tests repeated after 6 months from delivery were negative.

**Table 2:** Concordance of HPV genotypes in mother-neonate pairs

**Participants**	**Mother HPV**	**Newborn HPV**	
	**Genital**	**Oral** **at day 0**	**Oral** **at 6 months**
4	11, 56	11	-
12	53, 66	53	-
13	40, 44, 53	40, 44, 53	-
42	35, 33	35	-
51	58, 31, 68	58	-
58	others	Others	-
113	56	56	-
151	16	16	-
177	16	16	-
201	6	6	-

### Factors associated with vertical transmission

To find out the reproductive characteristics contributing to vertical transmission of HPV, the pairs with HPV-positive mothers were allocated into 2 groups according to HPV status of newborns (Table 3). Neonates had greater chances of acquiring infection when their mothers were infected with multiple types (p = 0.003). Among 10 infected newborns, all were delivered vaginally and none by caesarean section. The difference was not shown in terms of risk of HPV type, gestational age, birth weight, and genital infection. Length of labour and status of the amniotic membrane also showed the same values between HPV-positive and HPV-negative newborns.

**Table 3:** The reproductive characteristics associated with neonatal HPV status

**Characteristics**		**HPV(+)**	**(SD or %)**	**HPV(−)**	**(SD or %)**	**P-value**
Gestational age (w)		39.4	(0.9)	39.5	(1.0)	0.791*
Weight (g)		3260	(304)	3306	(328)	0.958*
Genital infection	(−)	10	(18.5)	44	(81.5)	1.000
	(+)	0		1		
Length of labour(min) (n = 45)		774	(424)	941	(622)	0.638*
PROM	(−)	6	(17.1)	29	(82.9)	1.000
	(+)	4	(20.0)	16	(80.0)	
Mode of delivery	CS	0	(0.0)	14	(100.0)	0.050
	VD	10	(24.4)	31	(75.6)	
Risk of HPV type	LR or other type	2	(8.7)	21	(91.3)	0.166
	HR type	8	(25.0)	24	(75.0)	
Number of HPV type	Single	5	(10.6)	42	(89.4)	0.003
	Multiple	5	(62.5)	3	(37.5)	

SD: standard deviation, PROM: premature rupture of membranes, CS: caesarean section, VD: vaginal delivery, LR: low risk, HR: high risk, P < 0.05 indicates statistical significance by a *t*-test* or a chi-square (or Fisher’s exact) test.

## Discussion and conclusions

The prevalence and concordance of HPV infection in mother neonate pairs by HPV genotype detection has been investigated. Comparison of HPV genotypes in relation to obstetric characteristics lead us to suggest the primary route of vertical transmission during perinatal period. According to epidemiological data of HPV infection in the female population, HPV prevalence ranges from 10 % in women with normal cytology to nearly 100 % in women with cervical cancer [3,12,13]. In addition to cervical disease, the age, region, HPV detection method and study design are also associated with the estimates of HPV infection [12].

For pregnant women, the percentage positivity varied from 5.5 to 65 % using the only meta-analysis to date [5]. Outstandingly high estimates tend to be reported by research conducted in an endemic area or for high risk participants identified with the pathos-mechanism of vertical transmission. Up to 60 % of young pregnant women were infected with HPV in Uganda, where a similar frequency was reported in non-pregnant women [14]. In contrast, a lower prevalence has been reported by large cohort studies of the general population. A recent study in Spain surveyed 828 women who were consecutively included at the perinatal clinic and found 6.5 % to be infected with HPV [15]. A study by Takakuwa et al. [16], one of the largest (n = 1183), presented an overall prevalence of 12.5 %, with the prevalence in women under 25 years of age being significantly higher compared than in women over 25 years (22.6 vs. 11.3 %, p < 0.0005).

The present study has shown the prevalence of HPV infection of the cervix to be 18.9 % in 291 Korean pregnant women without abnormal cervical lesion under colposcopy, which was comparable to those of large studies mentioned above. The infection was more frequently detected in women with abnormal cytology and without a history of parturition. Although statistically insignificant, young women under 30 years of age were more infected with HPV (Table 1).

Age is constantly reported to be related to the proportion of positive findings in pregnant women, as in non-pregnant women. Eppel et al. [17] showed decreased prevalence of HPV infection with increasing maternal age. The group younger than 30 years had twice the risk risk of the group older than 30 years. In young women, HPV infection is transient, which is considered to be one of the mechanisms explaining why the finding of HPV after the age of 25 or 30 years is less frequent [18].

Since Sedlacek et al. [5] detected HPV DNA in neonatal oropharyngeal aspirates [19], widely variable estimates of perinatal HPV infection have been reported in terms of neonatal prevalence, frequency of vertical transmission, and persistent infection. Their inconsistent results depend on selection criteria, sample size, period of follow-up and particularly the method of assay.

A higher rate of vertical transmission, 70-80 %, came from early small studies when analyzing methods for HPV DNA detection by PCR were still developing [9,20]. While PCR is known to be the most sensitive method and type-specific PCR can detect <10 copies of HPV DNA, this technique can give false-positives due to contamination, as it relies on a series of laboratory preparations for target amplification [21]. DNA sequencing, another highly sensitive method, can verify the genotype and reduce the probability of false-positives by sequencing. Smith et al. [6] were the first to sequence a number of HPV genotypes and reported that only 3.7 % (6/164) of neonates were positive for HPV DNA. However, this technique alone is unable to identify each viral type in multiple infections.

In our study, vertical transmission was found in 18.5 % (10/55) with HPV DNA chip, which can identify genotypes even in the cases with multiple infections through automated and efficient procedures. This technique is appropriate for large epidemiological studies and its efficacy for genotyping of HPV infection has been demonstrated in cervical cancer screening programs [10,11]. Our results are supported by the fact that research since 2000 has reported similar estimates ranging from 10-20 %, using strong laboratory methods that can genotype a number of HPVs [15,22,23].

We could add some data to the current consensus that HPV in a neonate was probably acquired during the passage through an infected cervix [5]. The genotypes were concordant in the mother newborn pairs with vertical transmission that made us infer the source of HPV infection (Table 2). In addition, 10 newborns with positive HPV were delivered vaginally (Table 3). Tenti et al. [8] reported very similar data that HPV DNA was positive only in babies delivered through the infected birth canal, and that the genotypes of the pairs were identical to each other.

However, many studies also presented biological evidence suggesting other possible routes of viral transmission. HPV DNAs have been detected in sperm, amniotic fluid, placenta, cord and maternal blood [24-26]. Further, the concordance of HPV types between mothers and newborns is 57-69 % and HPV-positive newborns having HPV-negative mothers have been reported [2]. These results propose that some newborns can acquire HPV DNA through horizontal transmission from other contact after birth or in uterine transmission at an untested interval during early pregnancy.

In pregnant women, a few studies have assessed the extent of multiple infections, which is considered uncommon. With the introduction of laboratory techniques that can simultaneously identify multiple types of HPV, some investigators have reported that co-infection was detected in 17-66 % of the infected women [15,22,23]. Several studies have attempted to picture the association of multiple infections with vertical transmission. Tseng et al. [27] could distinguish only 2 types (HPV-16, -18) with PCR and reported that vertical transmission developed in 4 out of 11 women with double infection and 23 out of 57 with single infections. Rombaldi et al. [23] used the novel nested multiplex PCR with an ability to differentiate 19 types and showed that each 4 cases of vertical transmission occurred in 19 pairs with single infection and 30 with multiple infections. In this report, we demonstrated that multiple infection had a significantly higher probability of transmitting to newborns than a single infection (5/8 vs. 5/47, p = 0.003) (Table 3).

It has been argued whether or not the detection of HPV DNA after birth reflects a true infection or contamination. Because HPV infection has a long latent period, the repeated detection of HPV in the same area is considered as persistent infection. Some early studies used PCR and serially detected HPV DNA in the buccal and genital swabs at birth and at weeks to months of postpartum. Cason et al. [9] demonstrated that HPV-16 DNA persisted for up to 6 months in 80 % of HPV positive infants at birth. Kaye et al. [28] showed HPV-16 in the samples by sequencing analysis from 13 maternal cervices, and from their infants at 6 weeks and 2 years of age. The concordance was estimated to be 69.2 % and at least 15.4 % of infants maintained maternally-derived HPV infections until 2 years. In contrast to previous studies, we were unable to detect any persistent infection at 6 months in HPV positive newborns (Table 2). A prospective study reported that the virus was cleared in all infants as early as the 5th week during follow-up period until 18 months [8]. In a cohort study of Finnish families, the risk of infant HPV was not cervical HPV during pregnancy, but persistent maternal HPV infection [29]. These results indicate that vertical transmission at birth may not contribute to persistent infection in infants.

As commented in previous literature reviews, the estimates should be interpreted with caution in respect of geographic and demographic characteristics, research design, and detection methods of HPV [2,5,12]. Because our participants were consecutively included at one of the largest obstetric clinics in Korea where over 5000 newborns are delivered in a year, our estimates are presumed to have credable generality. In order to ascertain the persistency of HPV infection, we followed up the infants until 6 month postpartum, which was scarcely covered by previous studies [5,30]. We also discriminated over 24 HPV genotypes to evaluate the concordance with DNA chip, which is the one of the largest spectrums to date. Whilst HPV DNA chip is as efficient as the existing HPV detection methods, a recent study pointed out its limit of usefulness as a screening tool due to low sensitivity [31]. The combined use of advanced diagnostic procedures will allow a more accurate assessment of extent of HPV infection in pregnant women and newborns in further studies [32].

In conclusion, the infection and transmission of HPV was prevalent in pregnant women and their neonates. We suggest that the perinatal transmission of HPV takes place through an infected birth canal based on the concordance of HPV genotypes and the predominance of vaginal delivery. However, the clearance of HPV in all infants up to 6 months suggests that HPV DNAs of newborns do not indicate persistent infection in infants.

## Methods

This prospective study was approved by the institution’s ethics committee. Near term pregnant women anticipating deliveries at the department of obstetrics in a university affiliated hospital were recruited between April 2010 and January 2011. Participants fully understood the protocol of this study and signed written consent forms.

### Study subjects

Pregnant women over 36 weeks gestation were included. Exclusion criteria were as follows: severe intra-uterine growth restriction, severe oligohydroamnios or preterm premature rupture of membranes, severe hypertension during pregnancy, history of malignancies within 5 years, liver cirrhosis, renal failure, chronic diseases affecting maternofetal immunity and a history of psychological disease and alcoholism. On the day of admission for delivery, newborns of the participants were enrolled after maternal re-consent.

At enrolment, cervical swabs were taken of the pregnant participants. Exfoliative cervical cells were collected using cytobrush for HPV DNA chip analysis. Immediately after delivery, the oral mucosa of newborns was scraped for HPV DNA chip analysis. The test was repeated at 6 month postpartum for HPV-positive infants to identify persistent infection.

### HPV DNA genotyping

HPV detection and genotyping was performed using the HPV DNA Chip, PCR-based DNA microarray system, purchased from MyGene Company (Seoul, South Korea). The HPV DNA Chip contains 24 type specific probes; 15 types from the high-risk group (HPV-16, HPV-18, HPV-31, HPV-33, HPV-35, HPV-39, HPV-45, HPV-51, HPV-52, HPV-53, HPV-56, HPV-58, HPV-59, HPV-66, and HPV-68) and 9 types from the low-risk groups (HPV-6, HPV-11, HPV-34, HPV-40, HPV-42, HPV-43, HPV-44, HPV-54, and HPV-70). Briefly, DNA was isolated from swab samples using a DNA isolation kit (MyGene. Co, Seoul, Korea), and target L1 regions of HPV DNA were amplified using consensus GPd5+/GP6day + primers. Ten μlof the HPV-amplified product was denatured for 5 min at 95°C. The samples were mixed with a hybridization solution then applied onto the DNA Chip.

Hybridized HPV DNA was visualized using a DNA Chip scanner (Scanarray lite; GSI Lumonics®, Ottawa, Ontario, Canada). HPV amplicons can be hybridized with the corresponding type-specific oligonucleotide probe and visualized on HPV DNA Chip slides as double-positive spots when HPV DNA is present in amplified PCR product.

The samples that showed a positive band of 150 bp on the gel electrophoresis, but were negative on the HPV DNA Chip slide, were designated as HPV-other. None of the negative controls (without DNA) revealed HPV positivity.

### Statistical analysis

To identify the factors associated with HPV infection in pregnant women and neonates, participants were assigned into 2 groups according to the presence or absence of HPV. Continuous variables were compared with Student’s *t*-test and discrete with chi-square test or Fisher’s exact test. The Mantel-Haenszel test was used to control for the confounding from age to some factors which were significantly associated with maternal HPV status under univariate analyses. P < 0.05 was considered significant with the use of a 2-sided test.

## Competing interests

The authors declare that they have no competing interests.

## Authors’ contributions

HP and SWL participated in the study design and the statistical analysis, and drafted the manuscript. IHL and HMR contributed to acquisition and interpretation of the data. ARC and YSK carried out laboratory procedures and helped to draft the manuscript. SRH carried out cytological classification of specimens and participated in HPV genotyping. SSK and SJS contributed to the conception of the study and participated in the statistical analysis. SMS participated in the conception and design of the study and acquired the data of infants. TJK conceived of and designed the study, and co-ordinated the acquisition of the data and draft of the manuscript. All authors read and approved the final manuscript.
